# Mental health outcomes and alcohol consumption among UK military spouses/partners: a comparison with women in the general population

**DOI:** 10.1080/20008198.2019.1654781

**Published:** 2019-09-23

**Authors:** Rachael Gribble, Laura Goodwin, Nicola T. Fear

**Affiliations:** a King’s Centre for Military Health Research, Institute of Psychiatry, Psychology and Neuroscience, King’s College, London, UK; b Psychological Sciences, University of Liverpool, Liverpool, UK

**Keywords:** Military families, military spouses, women’s health, family health, mental health, depression, PTSD, alcohol consumption, familias de militares, cónyuges militares, salud de la mujer, salud familiar, salud mental, depresión, trastorno de estrés postraumático, consumo de alcohol, 军人家庭, 军人配偶, 女性健康, 家庭健康, 精神健康, 抑郁, 创伤后应激障碍, 饮酒量

## Abstract

**Background**: Military families can experience unique stressors that may contribute towards poorer well-being among the spouses/partners of Service personnel. However, there is little UK research regarding mental health or alcohol consumption among this population.

**Objective**: This study examined mental health outcomes (probable depression and post-traumatic stress disorder (PTSD)) and alcohol consumption among UK military spouses/partners compared to women in the general population. Associations with military and socio-demographic characteristics were examined.

**Method**: Survey data from 405 female spouses/partners of current and former UK Service personnel participating in a study of military-connected children (2010–2012) was analysed. Comparisons to women in the general population were made using the 2007 Adult Psychiatric Morbidity Survey (n = 1594).

**Results**: Compared to women from the general population, military spouses/partners were significantly more likely to meet criteria for probable depression (adj. OR 2.50 (95% CI 1.52–4.11)). There was no significant difference regarding probable PTSD. Spouses/partners were significantly more likely to meet criteria for hazardous alcohol consumption (adj. OR 2.55 (95% CI 1.87–3.47)) and more likely to report episodes of weekly, daily or almost daily binge-drinking (adj. OR 2.15 (95% CI 1.28–3.61)) than women in the general population. Binge-drinking was significantly higher among spouses/partners of Service personnel reporting family separations of more than 2 months in the last 2 years compared to those reporting no, or shorter, separations (adj. OR 1.88 (95% CI 1.08–3.27)).

**Conclusion**: This is the first study to examine mental health and alcohol consumption among UK military spouses/partners. The significantly higher prevalence of probable depression, hazardous alcohol consumption, and binge-drinking compared to women in the general population suggests further research is needed into the drivers of poor mental health and alcohol consumption among this population and in identifying or developing prevention campaigns to reduce alcohol use and support their well-being.

## HIGHLIGHTS

• There is little research on mental health or alcohol consumption among UK military spouses/partners• The prevalence of probable depression was significantly higher among spouses/partners compared to women in the general population.• Spouses/partners were also more likely to meet criteria for hazardous alcohol consumption and to report more episodes of binge-drinking.• The findings highlight the need for additional research on this population and to identify or develop prevention campaigns to reduce alcohol use and support well-being.

## Abstract (es)

**Antecedentes**: las familias de militares pueden experimentar factores estresantes únicos que pueden contribuir a un peor bienestar entre las esposas/parejas del personal del Servicio. Sin embargo, hay poca investigación en el Reino Unido sobre salud mental o consumo de alcohol en esta población.

**Objetivo**: Este estudio examinó los resultados de salud mental (depresión probable y trastorno de estrés postraumático (TEPT)) y el consumo de alcohol entre las esposas/parejas de militares del Reino Unido en comparación con las mujeres de la población general. Se examinaron las asociaciones con características militares y sociodemográficas.

**Método**: Se analizaron los datos de la encuesta de 405 esposas/parejas del personal actual y anterior del Servicio del Reino Unido que participaron en un estudio de niños vinculados al ejército (2010-2012). Las comparaciones con las mujeres en la población general se realizaron mediante la Encuesta de morbilidad psiquiátrica de adultos de 2007 (*N* = 1594).

**Resultados**: en comparación con las mujeres de la población general, las esposas/parejas de militares tenían significativamente más probabilidades de cumplir con los criterios de depresión probable (adj. *OR* 2.50 (IC 95% 1.52-4.11)). No hubo diferencias significativas con respecto al TEPT probable. Las esposas/parejas tenían muchas más probabilidades de cumplir con los criterios para el consumo de alcohol peligroso (adj. *OR* 2.55 (IC 95% 1.87-3.47)) y tenían más probabilidades de reportar episodios de consumo excesivo de alcohol semanal, diario o casi diario (adj. *OR* 2.15 (IC 95% 1.28-3.61)) que las mujeres en la población general. El consumo excesivo de alcohol fue significativamente mayor entre las esposas/parejas del personal del Servicio que informaron separaciones familiares de más de 2 meses en los últimos 2 años en comparación con las que informaron que no hubo separaciones, o más cortas (adj. *OR* 1.88 (IC 95% 1.08-3.27)) .

**Conclusión**: este es el primer estudio que examina la salud mental y el consumo de alcohol entre los cónyuges/parejas militares del Reino Unido. La prevalencia significativamente más alta de depresión probable, consumo de alcohol peligroso y consumo excesivo de alcohol en comparación con las mujeres en la población general sugiere que se necesita más investigación sobre los factores que conducen a la mala salud mental y el consumo de alcohol entre esta población y para identificar o desarrollar campañas de prevención para reducir consumo de alcohol y apoyo a su bienestar.

## Abstract (zh)

**背景：**军人家庭有时会经历独特的应激源，这可能导致服役人员的配偶或伴侣的身心健康状况更差。然而，英国很少有关于这一人群的心理健康或饮酒量的研究。

**目的：**本研究测量了英国军人配偶或伴侣相较于普通女性人群的心理健康结果（可能的抑郁症和创伤后应激障碍（PTSD））以及饮酒量。考查了与军事特征及社会人口学特征的关联。

**方法：**分析了来自405名参与了2010-2012年军队相关儿童研究的现任/前任英国服役人员的女性配偶或伴侣的调查数据。使用2007年成人精神疾病患病情况调查（n = 1594）中普通女性人群的数据进行了对比。

**结果：**与普通女性人群的相比，军人配偶或伴侣更有可能达到可能抑郁的标准（校正OR值为 2.50（95％置信区间为 1.52-4.11））。可能的创伤后应激障碍没有显著差异。军人配偶或伴侣比普通人群中的女性更有可能达到危险饮酒量标准（校正OR值为2.55（95％置信区间为1.87-3.47）），更有可能报告每周、每日发作或者几乎每日酗酒的情况（校正OR值为2.15（ 95％置信区间为 1.28-3.61)）。服役人员的配偶或伴侣中，报告了过去2年中有超过2个月与家人分离的，相较于报告没有家人分离或者分离时间更短的人相比，酗酒情况明显更高（校正OR值为1.88（95％置信区间为 1.08-3.27））。

**结论：**这是第一项考查英国军人配偶或伴侣心理健康和饮酒量的研究。此人群相较于普通人群中的女性，可能的抑郁症、有害饮酒和酗酒的流行率明显更高，这表明需要对此人群中心理健康变差和饮酒状况的驱动因素进行进一步研究，并确定或开展预防活动以减少酒精的使用，支持她们的福祉。

## 1. Introduction

As a ‘greedy institution’ (Segal, 1986) the military demands dedication and commitment from Service personnel. However, these demands are not just limited to those in military service. The families of Service personnel experience a number of unique stressors as a result of their husband or partner’s occupation, including regular relocation, family separation and reunion, and the involvement of Service personnel in operational deployments (Padden & Posey, 2013). Studies have demonstrated how these experiences can increase family stress and contribute to poor mental health and well-being among military spouses/partners (Burrell, Adams, Durand, & Castro, 2006; Drummet, Coleman, & Cable, 2003).

To date, there are no studies using quantitative methods to estimate mental health outcomes and alcohol consumption among UK military spouses/partners, with the majority of available research based on US data. While US research provides an understanding of the potential mental health problems among this population and the factors contributing to these outcomes, differences in both culture and military operations and structure mean that the findings cannot be assumed to apply to a UK context. For example, deployment lengths vary, with UK personnel deploying for 6 months under military harmony guidelines while US personnel can deploy for a year or longer (Fear et al., 2010). Prior UK research is largely qualitative, with studies highlighting how relocation, particularly overseas, and separation due to deployment can negatively affect spouse/partner well-being (Dandeker, French, Birtles, & Wessely, 2006; Gribble, Goodwin, Oram, & Fear, 2019; Higate & Cameron, 2004; Jervis, 2011; Quinault, 1992).

While interest in the health and well-being of military spouses increased following military operations in Iraq and Afghanistan, this population remains under-researched (De Burgh, White, Fear, & Iversen, 2011). The small body of literature in this area, combined with different research methodologies and study quality, has resulted in often varying findings regarding the mental health of military spouses/partners – prevalence estimates for depression range from 3–45% (Eaton et al., 2008; O’Toole, Outram, Catts, & Pierse, 2010; Renshaw, Rodrigues, & Jones, 2008), and post-traumatic stress disorder (PTSD) from 2–42% (Dursun & Sudom, 2009; Erbes, Meis, Polusny, & Arbisi, 2012; Renshaw et al., 2011). Few studies have examined alcohol consumption in this population (Gribble, Thandi, Goodwin, & Fear, 2017). This is despite high levels of alcohol use among Service personnel (Bray & Hourani, 2007; Fear et al., 2007; Jacobson et al., 2008) and known relationships between the drinking behaviours of couple members, especially for women (Demers, Bisson, & Palluy, 1999; Leonard & Homish, 2008). Studies comparing the mental health of military spouses/partners to women in the general population are also scarce, making it difficult to determine if there are additional or particular needs in this population. Those that have been conducted suggest significantly higher depression and PTSD among US and Australian spouses/partners compared to community samples, although similar levels of alcohol use (Alessi, Ray, Ray, & Stewart, 2001; Lester et al., 2010; O’Toole et al., 2010; Padden, Connors, & Agazio, 2011; Westerink & Giarratano, 1999).

Understanding how socio-demographic and military factors, such as deployment or differences in rank, may influence spouse/partner mental health and alcohol consumption is also unclear. Studies have identified associations between poorer spouse/partner mental health and spouse/partner unemployment, lower education, and shorter relationship duration (Dursun & Sudom, 2009; Herzog, Everson, & Whitworth, 2011; McGarigal, Jablonski, Ferri, & Lester, 2009; O’Toole et al., 2010). Associations with military factors include deployment, Service personnel rank, and increasing family separation (Faulk, Gloria, Cance, & Steinhardt, 2012; Kelley, 1994; Lester et al., 2010; Mansfield et al., 2010; Rosen, 1995). However, evidence in this area can be conflicting as many studies control for socio-demographic or military factors in analyses but do not examine the potential role they may play as independent risk factors.

To help address these gaps in the literature, this study examined the prevalence of mental health problems (probable depression, probable PTSD) and alcohol consumption among UK military spouses/partners compared to women in the general population. Associations between outcomes and socio-demographic and military factors were examined.

## 2. Methods

### 2.1. Description of samples

Two data sources were used. The first, the Children of Military Fathers study (Fear et al., 2018), was used to obtain data on military spouses/partners and the second, the 2007 Adult Psychiatric Morbidity Survey (APMS) (McManus, Meltzer, Brugha, Bebbington, & Jenkins, 2009), was used for data on women in the general population.

#### 2.1.1. Military spouses/partners

**Figure 1.**
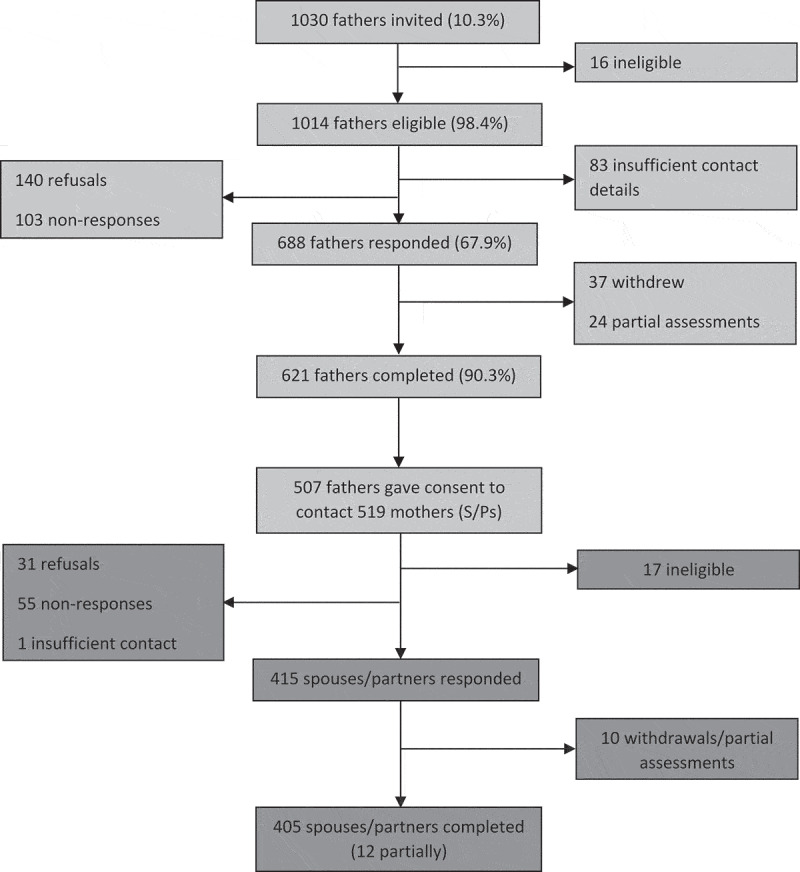
Flow diagram of Children of Military Fathers Study participants.

Military families were recruited into the Children of Military Fathers study via a step-wise approach using Service personnel as the key contact (Figure 1). Service personnel included in the Children of Military Fathers study were identified from the King’s Centre for Military Health Research (KCMHR) Health and Well-being cohort, a two-phase follow-up study of UK Armed Forces personnel established in 2003 (phase 1) to examine the health and well-being of personnel deployed to Iraq and Afghanistan (Hotopf et al., 2006). The phase 2 sample, replenished in 2007–2009 (Fear et al., 2010), was representative of the personnel structure of the deployable UK Armed Forces at the time of selection into the study (Defence Statistics, 2013).

Regular and reserve personnel at phase 2 of the KCMHR cohort were selected for participation into the Children of Military Fathers study if they reported having children aged 3–16 years and according to their score on the PTSD Checklist – Civilian Version (PCL-C) (Weathers, Litz, Herman, Huska, & Keane, 1993). Two groups were recruited. Group 1 was comprised of personnel with probable PTSD, defined as those who met PTSD caseness (score ≥50), borderline caseness (score 40–49) or who reported at least two of three symptom PCL-C PTSD cluster domains (n = 59). Group 2 was comprised of the remaining respondents. Of the 1030 Service personnel invited to participate based on these criteria, 931 were re-contacted, and 621 completed the survey (response rate 66.7%) (Fear et al., 2018).

Of the serving and ex-serving personnel who took part in the father’s component of the Children of Military Fathers study, 507 (81.6%) gave consent and contact details for the mothers of their children (Fear et al., 2018). As some personnel had children with multiple women, 519 current and former spouses/partners of Service personnel were contacted regarding participation. The final sample was comprised of 405 spouses/partners who completed (n = 393) or partially participated (n = 12) the survey (response rate 78.0%): all had at least one child. Of the 12 who partially participated, 11 did not complete mental health or alcohol measures. Four Service personnel had two spouses/partners – one current and one former. Both were included in all analyses.

Data was collected between July 2010 and October 2012. Ethical approval was granted by the Ministry of Defence Research Ethics Committee and the King’s College Hospital Research Ethics Committee (NHS REC reference: 08/H0808/27).

#### 2.1.2. Comparison sample

The 2007 Adult Psychiatric Morbidity Survey (APMS) (McManus et al., 2009) was chosen as the comparison sample as it is the largest community study of mental health in the UK and contains similar validated measures of mental health as those used in the Children of Military Fathers study. To improve comparability between the two studies, data from the APMS was restricted to women aged 25–55 years with at least one biological child in order to reflect a similar profile of women to that in the military spouses/partners sample. APMS respondents who had previously served in the Armed Forces or who were unsure of their serving status were excluded (n = 30). The final sample was composed of 1594 women.

### 2.2. Measures

#### 2.2.1. Mental health outcomes

##### 2.2.1.1. Probable depression

Probable depression among military spouses/partners was assessed using the Patient Health Questionnaire (PHQ-9), a 9-item measure of mood, concentration, sleeping, diet and behavioural symptoms in the previous 2-week period (Kroenke & Spitzer, 2002; Kroenke, Spitzer, & Williams, 2001; Löwe, Unutzer, Callahan, Perkins, & Kroenke, 2004). Each item is a 5-point Likert scale indicating how much participants have been bothered by certain problems in the last month, with response options from ‘Not at all (0)’ to ‘Nearly every day (3)’. Total scores range from 0 to 27, with higher scores indicating greater depression symptomology. It has been validated in primary care samples in the UK (Gilbody, Richards, & Barkham, 2007). Probable depression caseness was determined as a PHQ-9 score of ≥10, indicating moderate, moderately severe or severe depression (Kroenke & Spitzer, 2002).

The prevalence of depression among women in the APMS was determined as those meeting moderate or severe depression criteria as defined by the Clinical Interview Schedule-Revised (CIS-R) (Lewis, Pelosi, Araya, & Dunn, 1992). The CIS-R is an interviewer-administered structured interview covering non- psychotic symptoms in the prior week, such as somatic symptoms, fatigue, depression, anxiety and phobias. The CIS-R begins with two filter questions regarding symptomology, which are followed by questions regarding frequency, duration, severity of symptoms. Algorithmically derived ICD-10 diagnoses of moderate and severe depression were used to determined depression caseness (McManus et al., 2009).

##### 2.2.1.2. Probable post-traumatic stress disorder (PTSD)

Probable PTSD among military spouses/partners was assessed using the 17-item PTSD Checklist Civilian Version (PCL-C) (Weathers et al., 1993). Based on DSM-IV criteria, items cover repeated, disturbing memories thoughts or images of stressful events (re-experiencing), physical reactions to reminders of events (arousal) or avoiding stressful experiences or taking part in activities or situations that are reminders of traumatic events (avoidance/numbing). Each item is a 5-point Likert scale indicating how much participants were bothered by certain problems in the last month, with response options from ‘Not at all’ (1) to ‘Extremely’ (5). Total scores range from 17 to 85, with higher scores indicating greater PTSD symptomology. A PCL-C score of ≥44 was used in this study as it has been identified as the most appropriate for achieving high specificity and sensitivity among women in the general population (Terhakopian, Sinaii, Engel, Schnurr, & Hoge, 2008).

APMS respondents completed the Trauma Screening Questionnaire (TSQ) (Brewin et al., 2002). Caseness was determined by respondents experiencing a minimum of six out of ten re-experiencing and arousal items such as upsetting dreams about the event or irritability/outbursts of anger at least twice in the past week (yes/no responses). This measure does not include items on avoidance and numbing but has shown high sensitivity and specificity against the Structured Clinical Interview for DSM–IV (SCID) and the Clinically Administered PTSD Scale (CAPS) (Brewin et al., 2002).

#### 2.2.2. Alcohol consumption

The 10-item Alcohol Use Disorders Identification Test (AUDIT), a screening tool for identifying excessive or risky alcohol consumption and the consequences of harmful use (Babor, Higgins-Biddle, Saunders, & Monteiro, 2001), was used to determine alcohol consumption outcomes in both studies. The first 8-items are 5-point Likert scales with responses ranging from ‘Never’ (0) to ‘Daily or almost daily (4). Items 9 and 10 are 3-point Likert scales relating to alcohol-related injury and expressions of concern about drinking from others, with response ‘No’ (0), ‘Yes, but not in the last year’ (2), and ‘Yes, during the last year’ (4). Total scores range from 0 to 40, with higher scores indicating greater alcohol misuse.

Recommended cut-offs were used for alcohol misuse (AUDIT ≥8), hazardous alcohol consumption (score ≥1 on item 2 (number of drinks on typical day of drinking) or 3 (frequency of binge-drinking – ≥6 drinks per session)), possible dependence (score ≥1 on items 4–6 (inability to stop, needing a drink in morning, not performing usual tasks)) and harmful alcohol consumption – (score ≥1 on items 7–10 (guilt/remorse, injury to self or others, blackouts, concern about drinking from others)) (Babor et al., 2001). AUDIT items 1–3 (frequency of alcohol consumption, number of standard drinks per drinking session, and binge-drinking) were examined individually to compare alcohol consumption behaviours between military spouses/partners and women in the general population.

High internal consistency was shown within the sample of spouses/partners for the PHQ-9 (Cronbach’s α = 0.84), PCL-C (Cronbach’s α = 0.92) and AUDIT (Cronbach’s α = 0.99). Similar findings were found within the APMS data for CIS-R (Cronbach’s α = 0.98), TSQ ((Cronbach’s α = 0.99), and AUDIT (Cronbach’s α = 0.92).

#### 2.2.3. Socio-demographic and military characteristics

Military spouses/partners and APMS respondents provided socio-demographic information on age (years at time of survey), relationship status, and number of children (Table 1). Information on military spouse/partner occupation was used to determine occupational social class in accordance with the National Statistics Socio-economic Classification (NS-SEC), a standardized method of classifying occupations within the UK according to the level and content of skill involved in each job (managerial/professional, intermediate and routine/manual, and unemployed/never worked) (Office for National Statistics, 2010). APMS NS-SEC occupational social class was provided with the data. Additional socio-demographic information provided only by military spouses/partners included self-reported proximity to military bases, current postcode (used to derive urban/rural residence via the Rural-Urban Definition for Small Area Geographies method (RUC2011) (Bibby & Brindley, 2013), and age of youngest child (years).

Information collected from Service personnel in the Children of Military Fathers study was used to examine associations between military factors and spouse/partner mental health. These included Service (Royal Navy, Royal Marines, Army, Royal Air Force), rank (officer, non-commissioned officer (NCO), other ranks (corporal or lower)), engagement type (regular or reserve), current or former serving member of the UK Armed Forces, length of service (years), combat role in parent unit, experience of deployment to Iraq and/or Afghanistan, and self-reported separation from children in the last 2 years (Table 1).

**Table 1.** Socio-demographic and military affiliations of military spouses/partners compared to women in the APMS and the KCMHR cohort study.

Socio-demographic characteristics	S/Ps % (n)	APMS % (n)	X^2^	df	p value
**Age (years)**	**N = 405**	**N = 1594**			
25–34	27.5 (100)	24.6 (386)			
35–44	50.7 (202)	39.4 (644)			
45–55	21.8 (90)	36.0 (564)	12.25	2	<0.001
**Relationship status**					
Married	86.4 (337)	66.2 (912)			
Cohabiting	10.2 (28)	12.0 (168)			
Other	3.4 (11)	21.9 (514)	23.59	2	<0.001
**Number of children**					
1	49.5 (192)	25.6 (419)			
2+	50.5 (213)	74.4 (1175)	42.49	1	<0.001
**Social occupational class**					
Managerial/professional	24.1 (88)	33.0 (494)			
Intermediate	28.7 (106)	17.6 (261)			
Routine/manual	17.5 (62)	27.1 (414)			
Unemployed/never worked	29.7 (113)	22.4 (370)	12.20	3	<0.001
Personnel military characteristics	S/Ps % (n)	KCMHR cohort % (n)	X^2^	df	p value
**Service**					
Royal Navy	13.1 (50)	13.3 (1008)			
Royal Marines	5.6 (21)	3.1 (350)			
Army	64.4 (260)	64.3 (5764)			
Royal Air Force	16.9 (74)	19.3 (1677)	2.25	3	0.083
**Rank**					
Officers	19.9 (106)	19.2 (1869)			
NCOs	68.1 (254)	61.3 (4902)			
Other ranks	12.0 (45)	19.5 (2028)	6.72	2	0.001
**Engagement type**					
Regular	86.9 (348)	90.1 (7413)			
Reserve	13.1 (56)	9.9 (1386)	4.30	1	0.038
**Serving status**					
Still serving	61.9 (255)	73.6 (6799)			
No longer in Service	38.1 (150)	26.4 (1979)	21.47	1	<0.001
**Length of service**					
0–9 years	15.0 (57)	34.3 (3494)			
10–22 years	55.3 (207)	44.5 (3401)			
>22years	29.8 (127)	21.2 (1588)	28.39	2	<0.001
**Experience of combat role**					
No combat role	79.4 (326)	76.8 (6728)			
Combat role	20.6 (79)	23.2 (2071)	1.15	1	0.283
**Experience of Iraq/Afghanistan deployment**					
No deployment	39.7 (170)	38.5 (2580)			
Deployed	60.3 (235)	61.5 (6219)	0.20	1	0.652
**Experience of family separation**					
No experience	24.7 (101)	–			
>1 month	34.8 (150)	–			
2–4 months	20.8 (81)	–			
> 5 months	19.7 (67)	–	–	–	–

Missing n = 13–502.

Due to the low numbers within some socio-demographic and military factors (n < 50), variables were largely analysed as binary using appropriate cut-offs (e.g. age of youngest child was collapsed into primary (3–10 yrs) or secondary (11–18 yrs)).

## 3. Statistical analyses

As no information was available on the characteristics of military spouses/partners who did not respond, inverse probability response weights (Mansournia & Altman, 2016) were generated according to the characteristics of the Service personnel they were in a current or former relationship with at the time of the Children of Military Fathers study. Response among military spouses/partners was predicted by Service personnel rank, number of mothers per Service personnel, relationship status, and Service personnel experience of deployment to Iraq and/or Afghanistan (Gribble, 2017). APMS response weights were supplied with the data.

Analyses were undertaken using Stata© 14.2 (StataCorp, 2015). The socio-demographic profile of the military spouse/partner sample is presented alongside that of participants in the APMS survey and KCMHR cohort in order to understand the comparability of samples to women from the general population and to a representative, deployable military population (Table 1). The prevalence of probable depression, probable PTSD, alcohol misuse, and alcohol consumption behaviours among military spouses/partners and women in the general population were estimated using weighted percentages presented alongside unweighted cell counts. Comparisons between prevalence estimates of military spouses/partners and women in the general population were conducted using logistic regression (Table 2). As there were a low number of cases of probable depression and probable PTSD among military spouses/partners (n < 30), comparisons were adjusted for respondent age only. Comparisons of alcohol misuse and alcohol consumption behaviours were adjusted for variables associated with alcohol use and common to both studies – age, occupational social class, and number of children (Fone, Farewell, White, Lyons, & Dunstan, 2013; Health and Social Care Information Centre, 2013; Maloney, Hutchinson, Burns, & Mattick, 2010).

**Table 2.** Prevalence of mental health and alcohol outcomes among military spouses/partners compared to women in the general population (%, N, unadj., adj. ORs).

Mental health outcomes	S/Ps % (N) N = 405	APMS % (N) N = 1594	Unadj. OR (95% CI)^a^	Adj. OR (95% CI)	p value
**Probable depression (PHQ-9)**					
No/mild depression	92.9 (366)	96.8 (1531)	1.0	1.0^b^	
Moderate/severe depression	7.2 (28)	3.2 (63)	2.34 (1.43–3.82)	2.50 (1.52–4.11)	<0.001
**Probable PTSD (PCL-C)**					
No (<44)	93.6 (374)	95.9 (1504)	1.0	–	
Yes (≥44)	6.4 (20)	4.1 (71)	1.60 (0.81–3.16)	–	
**AUDIT alcohol misuse outcomes**					
**AUDIT score categories**					
Little or no risk (≥7)	84.6 (337)	86.2 (1355)	1.0	–	
Medium risk (8–15)/high risk/probable dependence (≥16)	15.4 (57)	13.8 (239)	1.14 (0.81–1.60)	–	
**AUDIT subscales – item thresholds**					
**Hazardous alcohol consumption (AUDIT 2–3)**					
No hazardous use (0)	21.6 (80)	40.4 (501)	1.0	1.0^c^	
Probable hazardous use (≥1)	78.4 (270)	59.6 (795)	2.47 (1.85–3.29)	2.55 (1.87–3.47)	<0.001
**Alcohol dependence (AUDIT 4–6)**					
No dependence (0)	84.9 (301)	84.2 (819)	1.0	–	
Probable alcohol dependence (≥1)	15.1 (49)	15.8 (160)	0.95 (0.65–1.39)	–	
**Alcohol-related harm (AUDIT 7–10)**					
No alcohol-related harm (0)	73.6 (262)	69.5 (669)	1.0	–	
Probable alcohol-related harm (≥1)	26.4 (88)	30.5 (310)	0.82 (0.61–1.10)	–	
**Individual AUDIT questions (AUDIT 1–3)**					
**Frequency of alcoholic drink in past year**					
Never-monthly	39.2 (147)	40.0 (429)	1.0	1.0^c^	
2–4 times/month	29.4 (118)	26.8 (381)	0.87 (0.64–1.17)	0.81 (0.58–1.14)	0.224
2–3 times/week or more	31.4 (129)	42.2 (598)	0.59 (0.44–0.79)	0.57 (0.41–0.78)	0.001
**Number of standard drinks per typical session**					
1–2	45.6 (165)	63.3 (793)	1.0	1.0^c^	
3 or more	54.4 (185)	36.7 (503)	2.06 (1.60–2.65)	2.15 (1.64–2.81)	<0.001
**Binge-drinking – ≥6 units on one occasion**					
Never	25.3 (95)	46.0 (576)	1.0	1.0^c^	
<Monthly, monthly	65.0 (224)	45.1 (599)	2.62 (1.98–3.46)	2.54 (1.88–3.43)	<0.001
Weekly, daily, almost daily	9.7 (31)	8.9 (124)	1.98 (1.22–3.21)	2.15 (1.28–3.61)	0.004

Missing = 11–615.

^a^Baseline data APMS, no caseness/little or no risk/never-monthly frequency of alcohol use/1–2 std drinks/never binge-drink.

^b^Adjusted for S/P, APMS participant age.

^c^Adjusted for S/P, APMS participant age number of children (1, 2+) & occupational social class (managerial/professional (baseline), intermediate, routine/manual, unemployed/never worked.

Differences in outcomes between groups of military spouses/partners were examined through associations between spouse/partner mental health and alcohol consumption, spouse/partner socio-demographics and Service personnel military factors. Due to the low number of cases of probable depression and probable PTSD among military spouses/partners, associations were examined using unadjusted and adjusted negative binomial regression models of PHQ-9 and PCL-C measure scores to estimate incidence rate ratios (IRRs) (Table 3). Associations with binge-drinking were examined using unadjusted and adjusted logistic regression analyses estimating odds ratios (ORs) (Table 4). For both negative binomial and logistic regression analyses, two sets of multivariable models were developed using a value of p < 0.10 to identify variables for inclusion (Bursac, Gauss, Williams, & Hosmer, 2008) and ensure model parsimoniousity. The first included spouse/partner socio-demographic characteristics significant at p < 0.10 in univariable regressions and the second included Service personnel military characteristics significant at p < 0.10 in univariable regressions. Spouse/partner age, defined as an a priori covariate, was included in both models given the association with mental health outcomes (McManus et al., 2009). A significance level of p < 0.05 was used to identify variables associated with the outcomes of interest in the final models.

**Table 3.** Associations between military spouse/partner PHQ-9 and PCL-C scores, military spouse/partner socio-demographics and Service personnel military characteristics (IRR).

	Unadj. PHQ-9 score IRR (95% CI)^a^	Adj. PHQ-9 score IRR (95% CI)	p value
**S/P socio-demographic variables**			
S/P age	1.16 (0.95 − 1.43)	1.12 (0.93–1.35)^b^	0.215
Employed outside the home	0.76 (0.57–1.01)	0.87 (0.68–1.11)^b^	0.255
Proximity to military base	1.23 (0.98 − 1.57)	1.24 (0.97–1.60)^b^	0.091
Urban/rural residence	0.92 (0.67–1.26)	–	
No. of children	0.90 (0.69–1.17)	–	
Age of youngest child	1.21 (0.94–1.55)	–	
Relationship status	0.87 (0.55–1.37)	–	
Occupational social class		–	
*Managerial & Professional/Intermediate*	1.0	–	
*Routine & manual*	1.18 (0.86–1.61)	–	
*Unemployed*	1.30 (0.94–1.80)	–	
**Service personnel military variables**		–	
Service	1.19 (0.90–1.57)	–	
Rank	1.13 (0.88–1.46)	–	
Engagement type	0.93 (0.68–1.28)	–	
Serving status	1.09 (0.82–1.45)	–	
Length of service	1.04 (0.88–1.22)	–	
Combat role	0.76 (0.56–1.04)	0.78 (0.58–1.05)	0.104
Experience of deployment	0.81 (0.63–1.06)	–	
Family separation	0.92 (0.71–1.18)	–	

Missing = 1–115 ‡p < 0.10 *p < 0.05 **p < 0.01 ***p < 0.001.

^a^Baseline – S/P characteristics = Age 25–34 years, not employed outside the home, residing in town/village away from base, residing in major/minor urban/city/town, having 1 child, youngest child aged 3–10 years, in married relationship, managerial & professional occupational social class; Service personnel characteristics = Army, officer rank, regular engagement type, still serving, 10–22 years of Service, no combat experience, no experience of deployment to Iraq/Afghanistan, no experience of family separation in last 2 years.

^b^Adjusted for S/P age, S/P employment & S/P self-reported proximity to military base.

^c^Adjusted for S/P age & Service personnel experience of combat role.

**Table 4.** Associations between binge-drinking among military spouse/partners and spouse/partner socio-demographics and Service personnel military characteristics (%, N, unadj., adj. ORs).

	Binge-drinking – ≥6 units on one occasion % (N)				
S/P socio-demographic variables	Never/<monthly	Monthly-almost daily	Unadj. OR (95% CI)^a^	Adj. OR (95% CI)	p value
**Age (years)**	74.4 (N = 267)	25.6 (N = 83)			
25–34	24.5 (58)	32.3 (25)	1.0	1.0^b^	
35–44	51.5 (138)	54.9 (45)	0.81 (0.43–1.51)	1.00 (0.47–2.14)	0.992
45–55	24.0 (69)	12.7 (13)	0.40 (0.18–0.89)	0.38 (0.14–1.07)	0.067
**Urban/rural residence**					
Major/minor urban, city/town	68.8 (132)	54.2 (31)	1.0	1.0^b^	
Rural town/village, dispersed	31.2 (63)	45.8 (25)	1.86 (0.96–3.60)	1.78 (0.92–3.45)	0.088
**Service personnel military variables**					
**Rank**					
NCOs/other	76.7 (188)	88.1 (69)	1.0	1.0^c^	
Officer	23.3 (79)	11.9 (14)	0.44 (0.23–0.84)	0.52 (0.26–1.01)	0.054
**Experience of combat role**					
No experience	81.0 (216)	70.5 (63)	1.0	1.0^d^	
Experience of combat role	19.0 (51)	29.5 (20)	1.78 (0.93–3.41)	1.67 (0.87–3.20)	0.123
**Experience of family separation**					
No experience	65.3 (178)	47.4 (41)	1.0	1.0^e^	
≥2 months	34.7 (86)	52.6 (41)	2.09 (1.21–3.59)	1.88 (1.08–3.27)	0.025

Missing = 0–72 ‡p < 0.10 *p < 0.05 **p < 0.01 ***p < 0.001.

^a^Baseline S/P age 25–34 years, residing in major/minor urban/city/town, officer rank, no experience of combat role by Service personnel, no experience of family separation in last 2 years by Service personnel.

^b^Adjusted for S/P age & S/P urban-rural residence.

^c^Adjusted for S/P age & Service personnel rank.

^d^Adjusted for S/P age & Service personnel experience of combat role.

^e^Adjusted for S/P age & Service personnel experience of family separation in last 2 years.

## 4. Results

### 4.1. Socio-demographic and military characteristics of military spouses/partners compared to women from the general population and the KCMHR cohort

Compared to women in the general population, military spouses/partners were more likely to be aged 35–44 years, to be married, to have one child, and to work in occupational roles with intermediate levels of skill (Table 1). Compared to the KCMHR cohort, military spouses/partners were more likely to be in a current or former relationship with personnel of non-commissioned officer rank, reservists, those who had left service, and had served 10 years or longer (Table 1).

### 4.2. Prevalence of mental health outcomes and alcohol consumption among military spouses/partners compared to women in the general population

Fewer than 8% of military spouses/partners met criteria for probable depression or probable PTSD (Table 2). Military spouses/partners were significantly more likely than women in the general population to meet criteria for probable depression after adjusting for age with a moderate effect size (adj. OR 2.50 (95% CI 1.52–4.11)). No significant difference was found in the prevalence of probable PTSD.

The majority of military spouses/partners (84.6%) did not meet criteria for alcohol misuse, alcohol dependence or alcohol-related harm (Table 2), however a moderate effect size was found for significantly higher hazardous alcohol consumption among military spouses/partners compared to women from the general population (adj. OR 2.55 (95% CI 1.87–3.47)). Analysis of alcohol consumption behaviours indicated that while military spouses/partners reported consuming alcohol significantly less frequently than women in the general population in the past year (adj. OR 0.57 (95% CI 0.41–0.78)), there was a moderate and significant increase in the number of standard drinks consumed per drinking session (adj. OR 2.15 (95% CI 1.64–2.81)) and significantly greater endorsement of periodic to almost daily binge-drinking (Table 2).

### 4.3. Socio-demographic and military associations with spouse/partner mental health outcomes and alcohol consumption

No significant associations were found between military spouse/partner scores for probable depression or probable PTSD and spouse/partner socio-demographic or Service personnel military characteristics in adjusted negative binomial regression models (Table 3).

Exploration of associations with alcohol consumption behaviours found a small effect for a significantly greater number of reported episodes of binge-drinking among military spouses/partners of Service personnel reporting family separation of longer than 2 months in the last 2 years compared to the spouses/partners of personnel who reported no or shorter periods of family separation after adjusting for age (adj. OR 1.88 (95% CI 1.08–3.27)) (Table 4). An association approaching significance was found between reduced military spouse/partner binge-drinking and Service personnel officer rank after adjusting for military spouse/partner age (adj. OR 0.52 (95% CI 0.26–1.01), p = 0.054).

## 5. Discussion

This study aimed to examine the prevalence of mental health outcomes (probable depression, probable PTSD) and alcohol consumption among UK military spouses/partners compared to women in the general population and to identify socio-demographic and military factors associated with these outcomes. The significantly higher prevalence of probable depression, hazardous alcohol consumption, and binge-drinking among UK military spouses/partners compared to women in the general population indicates there may be additional mental health needs and problematic drinking behaviours within this population that may be the result of exposure to Service life. No significant difference was found in the prevalence of probable PTSD or alcohol misuse between the two samples.

Although the prevalence of probable depression was significantly higher among UK spouses/partners than among women in the general population, the estimate was lower than those found in prior US research (7.2% vs. 12.2–15%) (Eaton et al., 2008; Erbes et al., 2012). While this suggests UK spouses/partners may have better mental health outcomes than their US counterparts, this may also be related to when data was collected. Most previous US studies were conducted during or close to periods of deployment and, therefore, possibly reflect heightened stress among spouses/partners at this time. Preliminary analyses of the Children of Military Fathers data identified few Service personnel who were deployed during or near the time data collection from spouses/partners. However, deployment to Iraq and Afghanistan was not associated with any of the mental health outcomes in this study, suggesting family experiences of these particular deployments may not have had an effect on military spouse/partner health and well-being.

The prevalence of probable PTSD was also lower than US estimates using the same PCL-C cut-off (6.4% vs. 12.5–30.5%) (Renshaw et al., 2011, 2008). Research has suggested PTSD may be transferred from Service personnel to their intimate partners (Dirkzwager, Bramsen, Adèr, & van der Ploeg, 2005), yet despite over-sampling personnel with borderline or probable PTSD caseness into the Children of Military Fathers study, there was no significant increase in the prevalence of probable PTSD among military spouses/partners compared to women in the general population. This indicates secondary PTSD may not be an important mental health issue within the UK military community.

Unlike probable depression and PTSD, alcohol misuse was higher among UK spouses/partners compared to those in the US (15.4% vs 3.0–10.7%) (Blow et al., 2013; Erbes et al., 2012; Gorman, Blow, Ames, & Reed, 2011), likely reflecting wider cultural variations in alcohol consumption (Organisation for Economic Co-operation and Development [OECD], 2010). However, findings from this study indicate alcohol behaviours may be poorer among military spouses/partners compared to other women in the UK as spouses/partners were significantly more likely to report binge-drinking and consumed a significantly higher number of alcoholic drinks when they did drink than women in the general population despite consuming alcohol significantly less often. The increased prevalence of binge-drinking among spouses/partners represents an important public health issue for the military community given the potentially adverse influences on physical and mental health arising from this pattern of drinking (Centers for Disease Control and Prevention [CDC], 2017; Public Health England, 2016)

Of particular note is the significant association between binge-drinking among spouses/partners and Service personnel separation from their children, and therefore the family, for two months or longer during the last 2 years. Given increases in alcohol consumption during times of excess stress (Keyes, Hatzenbuehler, & Hasin, 2011), this finding may reflect maladaptive coping strategies among spouses/partners during longer and repeated absences of Service personnel from home (Gribble & Fear, 2019). Probable depression or probable PTSD measure scores were not found to be significantly associated with spouse/partner socio-demographics or Service personnel military characteristics, although some were approaching significance. This is likely to be due to the low number of participants meeting caseness criteria for these outcomes as well as the timing of data collection in relation to key events such as combat deployments.

### 5.1. Strengths and limitations

This is the first UK study to provide estimates of mental health outcomes and alcohol consumption among UK military spouses and partners using quantitative methods. Validated measures were used to compare outcomes to women in the general population in order to assess the influence of military life on the mental health and well-being of spouses/partners. There was a high response rate for military spouses/partners in this study (78.0%), reducing the possibility of non-response bias in this sample and increasing the reliability of the reported findings. The majority of the significant associations show a moderate effect size and reflect general findings from prior research in this area.

There are limitations to this study that should be considered when reviewing the findings. As a cross-sectional study, prevalence estimates may vary across different time points and associations between outcomes and socio-demographic and military factors do not indicate a causal relationship. The nature of the Children of Military Fathers study and the method of recruitment may mean that the sample of spouses/partners is not representative of the wider community. While the exact size of the UK military community is uncertain, 31% of Service personnel stated they have one or more children and 44% of regular UK Service personnel reported they were married or in a civil partnership near the time the survey was conducted (Head of Defence Statistics (Tri Service), 2014). The findings of this study are therefore likely to reflect nearly a third of UK military spouses/partners, a sizeable proportion of this under-researched population. Future studies could address these limitations by using longitudinal cohort studies to examine variation in outcomes over time to determine how the mental health of military spouses/partners changes from entry into the military community, during their partner’s (or their own) military service and throughout the transition period. Larger studies should also be conducted to replicate and confirm the findings and include under-represented groups such as spouses/partners without children. This would allow for in-depth analysis of the prevalence of, and associations with, probable depression and probable PTSD in this population, which was limited in the current study by the low number of spouses/partners meeting caseness criteria for these outcomes.

Another potential limitation is the use of different measures of probable depression and probable PTSD in the Children of Military Fathers study and the APMS that may measure different conceptual aspects of mental health problems. Caution should therefore be applied in interpreting comparisons in prevalence estimates for these outcomes as well as to odds ratios demonstrating wide confidence intervals arising from low numbers of respondents meeting caseness criteria. Although the PHQ-9 was validated in the UK against a clinical interview tool (Gilbody et al., 2007), the comparison of prevalence estimates from a screening tool (PHQ-9) and a clinical interview (CIS-R) with greater sensitivity and specificity may partially explain the significant difference in the prevalence of probable depression between spouses/partners and women in the general population. Unlike the PCL-C, the TSQ does not include questions on avoidance/numbing and asks respondents to consider a different time frame for their PTSD symptoms. There are currently no studies comparing the PCL-C to the TSQ. However, the 2014 APMS used the PCL-C and reported a similar prevalence of probable PTSD among women aged 25 years and over to prior surveys employing the TSQ (McManus, Bebbington, Jenkins, & Brugha, 2016), suggesting we may expect no significant difference in the overall prevalence of probable PTSD when using these two measures.

The changing criteria for PTSD between DSM-IV and DSM-5 should also be considered (Regier, Kuhl, & Kupfer, 2013). While the changes to symptom away from anxiety-based disorders, and addition of specifiers within DSM-5 have provided greater clarity on the conceptual nature of PTSD (Pai, Suris, & North, 2017), its use instead of a DSM-IV-based measure may have resulted in a more conservative estimate of PTSD than presented if diagnostic criteria, or indeed ICD-11 criteria, had been utilised (Rosellini et al., 2015). However, given the potential for this population to experience indirect exposure to trauma via their partners’ military Service, the clarification around trauma exposure may provide more accurate estimates of PTSD. With estimates of caseness in this study determined by cut-off score, in order to monitor PTSD symptomology in this population over time future studies of mental health among this population should adopt a cut-off of ≥32 on the PCL-5 (Blevins, Weathers, Davis, Witte, & Domino, 2015; Hoge, Riviere, Wilk, Herrell, & FW., 2014).

### 5.2. Implications

The findings of this research indicate there may be a greater need for mental health and well-being support and services for UK military spouses and partners. Additional studies to confirm and understand the reasons for the significantly higher prevalence of depression in this population should be conducted, including an examination of key social and military factors associated with this outcome such as social support, deployment and family separation. Research should also be undertaken to identify the drivers of hazardous alcohol consumption and binge-drinking among military spouses and partners and determine whether this more harmful pattern of alcohol consumption illustrates a transfer of cultural norms regarding alcohol use within the military community from Service personnel to their female spouses/partners and how it may affect other family members (Fear et al., 2007; House of Commons Welsh Affairs Select Committee, 2013).

Existing services and health care professionals within the NHS should be made aware of the potential for increased probable depression and alcohol consumption among this population and attempt to encourage spouses/partners to disclose any issues they may be experiencing. To aid this, health services should routinely collect data on whether someone is the spouse or partner of a serving or ex-serving member of the UK Armed Forces in order to better understand the outcomes of this population and target support where needed. Interventions to improve the mental health of spouses/partners should also be explored. These could be adapted from current programmes targeting alcohol consumption among serving personnel or from existing US programs specific to spouses/partners, such as US-based deployment programmes like ‘Families Over-Coming Under Stress’ (FOCUS) (Beardslee et al., 2011). The provision of online support and improving access to face-to-face services should also be explored to help identify ways to alleviate stress and anxiety that may arise as a result of military life, especially with the increasing trend towards greater geographical dispersal away from the military community on bases and the impact this can have on spouse/partner well-being (Gribble & Fear, 2019). Recent attempts to modify alcohol behaviours within the military community, including pilot studies of alcohol advisors to reduce consumption among military personnel (Ministry of Defence, 2015), should be widened to target improve the health and well-being of military families.

## 6. Conclusion

This is the first UK study to examine mental health outcomes and alcohol consumption among military spouses/partners compared with women in the general population. The significantly higher prevalence of probable depression, hazardous alcohol consumption, and binge-drinking among UK military spouses/partners compared to women in the general population indicates there may be additional mental health needs and problematic drinking behaviours within this population that may be the result of exposure to Service life. Additional research should be conducted to better understand the drivers of poor mental health and greater alcohol consumption among military spouses/partners and to identify or develop programs to support spouse/partner mental health and well-being.
